# Adipose-derived mesenchymal stem cell therapy for reverse bleomycin-induced experimental pulmonary fibrosis

**DOI:** 10.1038/s41598-023-40531-9

**Published:** 2023-08-14

**Authors:** Xiansheng Zhao, Jinyan Wu, Ruoyue Yuan, Yue Li, Quyang Yang, Baojin Wu, Xiaowen Zhai, Jiucun Wang, Jérémy Magalon, Florence Sabatier, Aurélie Daumas, Winston M. Zhu, Ningwen Zhu

**Affiliations:** 1 Department of Dermatology, Huashan Hospital, Fudan University, Shanghai, 200040 China; 2 Department of Plastic, Reconstructive and Burns Surgery, Huashan Hospital, Fudan University, Shanghai, 200040 China; 3 Children’s Hospital of Fudan University, Shanghai, China; 4 State Key Laboratory of Genetic Engineering, Collaborative Innovation Center for Genetics and Development, School of Life Sciences, Fudan University, Shanghai, China; 5 Culture and Cell Therapy Laboratory, INSERM CIC BT 1409, Assistance Publique Hôpitaux de Marseille (AP-HM), Aix-Marseille University, Marseille, France; 6 Aix Marseille University, INSERM, INRA, C2VN, Marseille, France; 7 Internal Medicine Department, Assistance Publique Hôpitaux de Marseille (AP-HM), Marseille, France; 8 Oxford Medical School, University of Oxford, Oxford, UK

**Keywords:** Mesenchymal stem cells, Mesenchymal stem cells

## Abstract

Idiopathic pulmonary fibrosis (IPF) is a chronic, progressive respiratory disease. Arguably, the complex interplay between immune cell subsets, coupled with an incomplete understanding of disease pathophysiology, has hindered the development of successful therapies. Despite efforts to understand its pathophysiology and develop effective treatments, IPF remains a fatal disease, necessitating the exploration of new treatment options. Mesenchymal stromal/stem cell (MSC) therapy has shown promise in experimental models of IPF, but further investigation is needed to understand its therapeutic effect. This study aimed to assess the therapeutic effect of adipose-derived mesenchymal stem cells in a bleomycin-induced pulmonary fibrosis model. First, MSC cells were obtained from mice and characterized using flow cytometry and cell differentiation culture methods. Then adult C57BL/6 mice were exposed to endotracheal instillation of bleomycin and concurrently treated with MSCs for reversal models on day 14. Experimental groups were evaluated on days 14, 21, or 28. Additionally, lung fibroblasts challenged with TGF-β1 were treated with MSCs supernatant or MSCs to explore the mechanisms underlying of pulmonary fibrosis reversal. Mesenchymal stem cells were successfully isolated from mouse adipose tissue and characterized based on their differentiation ability and cell phenotype. The presence of MSCs or their supernatant stimulated the proliferation and migration of lung fibrotic cells. MSCs supernatant reduced lung collagen deposition, improved the Ashcroft score and reduced the gene and protein expression of lung fibrosis-related substances. Bleomycin-challenged mice exhibited severe septal thickening and prominent fibrosis, which was effectively reversed by MSCs treatment. MSC supernatant could suppress the TGF-β1/Smad signaling pathway and supernatant promotes fibroblast autophagy. In summary, this study demonstrates that MSCs supernatant treatment is as effective as MSCs in revert the core features of bleomycin-induced pulmonary fibrosis. The current study has demonstrated that MSCs supernatant alleviates the BLM-induced pulmonary fibrosis in vivo. In vitro experiments further reveal that MSC supernatant could suppress the TGF-β1/Smad signaling pathway to inhibit the TGF-β1-induced fibroblast activation, and promotes fibroblast autophagy by Regulating p62 expression. These findings contribute to the growing body of evidence supporting the therapeutic application of MSCs in cell therapy medicine for IPF.

## Introduction

Idiopathic pulmonary fibrosis (IPF) is a chronic progressive respiratory disease characterized by clinical features such as shortness of breath, hypoxemia, and prominent lung infiltrates on imaging^1^. The exact etiology of IPF is unknown, but it is thought to result from the interaction between environmental exposures and genetic predisposition^2–4^. At present, the pathogenesis of pulmonary fibrosis is not clear and there is no effective therapeutic measure available to control the progression of the disease^5^. Despite numerous clinical trials, there are no FDA-approved therapies for IPF. Despite advancements in the understanding of IPF, effective treatment for this fatal disease are still lacking. Current therapies aim to slow disease progression rather than reverse it, with limited success^6^.

Recent studies have revealed that the development of IPF is closely linked to aberrant repair processes of alveolar epithelial cells following injury^7^. Prolonged stimulation of alveolar epithelial cells or exposure to a chronic inflammatory environment enhances their interaction with lung interstitial cells. The interaction leads to the recruitment of interstitial cells in the alveolar area, resulting in excessive extracellular matrix proliferation, thickening of the alveolar walls, and reduced lung compliance, ultimately causing progressive and irreversible damage^8^. The persistence of fibrotic accumulation represents the end stage of this pulmonary inflammatory disease^9,10^.

The complex interplay between different subsets of immune cell and the incomplete understanding of IPF's pathophysiology have contributed to the lack of successful therapies. Despite extensive efforts to unravel the underlying mechanisms of IPF and identify therapeutic targets, most of these targets have not translated into successful treatments^11,12^. Therefore, there is an urgent need for novel therapies for IPF.

With the advancement of treatment strategies, cell-based therapies have emerged as a promising option for various diseases^13^, including pulmonary fibrosis^14^. Research findings indicate that stem cells play an important role in repairing pulmonary tissue damage^15^. Stem cells are attracting growing attention in the field of regenerative medicine, providing new ideas for treating IPF with transplanted stem cells^14^. Although there is a wide range of stem cells currently used for IPF treatment, including lung-derived stem cells, bone marrow, fat, placenta, and other sources of mesenchymal stem cells, this study chose the adipose-derived MSCs owing to its strong differentiation ability, low immunogenicity, and no ethical conflicts. Adipose-derived MSCs have gradually become the "seed cell" in the research of IPF stem cell therapy^16^.

Among these therapies, adipose-derived MSC therapy has shown particular promise^17,18^. Studies have shown that MSCs possess immune regulatory and tissue repair functions^19,20^. They can secrete anti-inflammatory factors, thereby reducing the risk of cytokine storm and acute respiratory distress syndrome (ARDS), which consequently decreases mortality in critically ill patients^21^. Additionally, MSCs can secrete trophic factors and exhibit multidirectional differentiation ability, enabling them to repair lung tissue damage, and prevent or reverse the process associated with refractory lung injury caused by viral pneumonia^22^. Previous studies have highlighted the potential of MSC therapy in preventing or reversing fibrotic in various organs, including the lungs. In the context of IPF, MSC therapy has demonstrated promising results in preclinical studies, such as reducing inflammation, improving lung function, and reversing fibrosis^23^.

The objective of this study was to investigate the therapeutic effect of adipose-derived MSCs in a bleomycin-induced pulmonary fibrosis model. This study hypothesized that MSC therapy could prevent and reverse the core features of IPF, and that the beneficial effects of MSCs may be mediated through their paracrine-related cytokines. Overall, This findings provide further evidence for the significance of MSCs in cell therapy medicine applications for IPF treatment. Understanding the mechanisms underlying the therapeutic effects of MSCs in IPF could pave the way for the development of more effective and targeted therapies for this devastating disease.

## Methods

### Animal ethics approval

C57BL/6 mice were obtained from the Fudan University Animal Center.

All animals were placed in stainless steel cages in a well-ventilated room maintained at an ambient temperature of 24–26 °C with controllde relative humidity. Throughout the experiment, the animals under trial were provided with diet pellets and tap water. At the end of experimental period, all animals were euthanized under mild anesthesia by 20 mg/kg of ketamine and 3 mg/kg of xylazine. All animal experiments were performed with the approval of the Scientific Investigation Board of Fudan University (2019-JS-011). The study is reported in accordance with ARRIVE guidelines (https://arriveguidelines.org). All methods were performed in accordance with the relevant guidelines and regulations.

### Bleomycin-induced pulmonary fibrosis model

Female or male 8-week-old C57BL/6 mice (Shanghai, China) were anesthetized with isoflurane and received a single endotracheal dose of bleomycin sulphate (50 μL, 3 U/kg) at day 0 to induce pulmonary fibrosis. Bleomycin naive mice (control) received an endotracheal dose of saline (50 μL). At day 14, the treated group received a single intravenous (tail vein) dose of MSC (200 μL; dose, 5 × 10^6^ MSCs). The control group received an equivalent volume of phosphate-buffered saline (PBS). Mice were assessed at day 14 and/or at day 21 or day 28 for cytometric, histological, and/or quantitative PCR (qPCR) analysis.

### Isolation and culture of MSCs and lung fibroblasts

MSCs and lung fibroblasts were obtained from C57BL/6 mice. All procedures for the isolation and culture these cells were performed with the approval of the Scientific Investigation Board of Fudan University.

The male mice was euthanized under mild anesthesia by 20 mg/kg of ketamine and 3 mg/kg of xylazine. Adipose tissue was obtained from the epididymis using surgical manipulation, and blood vessels were separated from the fat and cut into 1 mm^3^ on a cold plate. The tissue was then digested using in 1 mg/mL collagenase I (Biosharp, China) at 37 °C for 0.5 h to obtain a single-cell suspension. The suspension was centrifuged at 2000 rpm for 5 min, and the cells were plated in a 100 mm dish. The culture medium consisted of Dulbecco's modified Eagle's medium, which contains 10% FBS, 100 mM non-essential amino acids, and 100 mM sodium pyruvate, 20 mM sodium pyruvate, β-mercaptoethanol L-glutamine, 20 μg/mL EGF, 100 IU/mL penicillin and 100 μg/mL streptomycin (all from Gibco, Carlsbad, CA). After that, the MSCs were cultured at 37 °C and 5% CO_2_ for about 5 days. When the cells reach 70–80% confluence, they were harvested using 0.05% trypsin (Gibco) and subcultured in the same medium. All experiments were performed using MSCs at the third generation.

The lung tissue of mice was cut into 1 mm^3^ piece under a sterile environment, and then placed in a H-glutamine, 20 μg/mL EGF, 100 IU/mL penicillin, and 100 μg/mL streptomycin (all from Gibco, Carlsbad, CA). After that, Lung tissue was cultured at 37 °C and 5% CO_2_ for about 7 days. When the cells reach 80% confluence, they were harvested using 0.05% trypsin (Gibco) and subcultured in the same medium. All experiments were performed using lung fibroblasts at the third generation.

### Flow cytometry analysis

To confirm the identity of the MSCs, flow cytometry analysis was performed according to the guidelines set by the International Society for Cellular Therapy (ISCT). has defined that the MSCs can express the surface markers CD29, CD44, CD73, and CD90 but lack of expression of CD34, CD45, CD11b^24–26^. MSCs at the third passage were trypsinized, washed twice with PBS and every 1 × 10^5^ cells were stained separately with human monoclonal antibodies, including CD29-FITC, CD44-FITC, CD90-FITC, CD45-FITC and CD11b-FITC (BD Biosciences, Franklin Lakes, NJ). Cells were incubated with the antibodies at 4 °C in the dark for 20 min, along with a fluorescein isothiocyanate (FITC) isotype control. After washing, the cells were suspended in 500 μL of FACS buffer and analyzed by Flow Cytometer (Accuri C6, BD Biosciences).

### MSC cell differentiation identification

To determine the multi-lineage differentiation potentiality of the MSCs, adipogenic, osteogenic, and chondrogenic differentiation experiments were performed using the Mesenchymal Stem Cell Functional Identification Kit (R&D Systems Inc., Minneapolis, MN). The induction processes for the three lineages were carried out according to the manufacturer’s instructions, with differentiation media changed every two days. After 10 days, the adipogenic, osteogenic and chondrogenic differentiation were sssessed using oil red staining, Alizarin Red-S staining, and alcian 8GX blue staining (Sigma-Aldrich, St. Louis, MO), respectively.

### Cell fluorescent labeling

For cell visualization, the MSCs were labeled with PKH26 Red Fluorescent Cell Connection Kit (Tanon; Tanon Science & Technology, Shanghai, China) to stain the cell membrane, and DAPI was used to stain the nucleus. A total of 2 × 10^7^ MSC cells were stained following the instructions provided with the kit. Cell images were captured with a laser scanning confocal microscopy (Olympus, Tokyo, Japan).

### RNA extraction and qRT-PCR analysis

Total RNA was extracted from tissues or cultured cells as previously described^27^. Total RNA was extracted by trizol according to kit instructions (Ambion, Foster City, CA). Then cDNA was synthesized using the All-In-One RT MasterMix (ABM). Forward and reverse primer sequences used in reverse transcriptase PCR (Table 1) were designed by Primer 5.0 software (PREMIER Biosoft International, Palo Alto, CA), based on mRNA information of each gene obtained from the National Center for Biotechnology Information (NCBI) database. Real-time PCR was performed using EvaGreen 2 × qPCR MasterMix (ABM) on a Light Cycler 480 II (Roche Diagnostics, Basel, Switzerland) with the designed forward and reverse primers.

**Table 1:** Reverse transcriptase PCR primers.

Primer name	Primer sequences (5′ to 3′)
TGF-β1-F	CTCCCGTGGCTTCTAGTGC
TGF-β1-R	CTCCCGTGGCTTCTAGTGC
Col I-F	GCTCCTCTTAGGGGCCACT
Col I-R	GCTCCTCTTAGGGGCCACT
a-SMA-F	CCCAACTGGGACCACATGG
a-SMA-R	TACATGCGGGGGACATTGAAG

### Western blotting analysis

A Western blot assay was performed as described^28^. For Western blot analysis of whole-cell extracts, the following primary antibodies were used: TGF-β1 (1:5000; Cell Signaling Technology, Danvers, MA, USA), α-SMA (1:5000; Abcam,), and Col I (1:5000; Abcam). Smad2 (1:1,000 dilution, Protein-Tech, China), phospho-Smad2 (1:1,000 dilution, Protein-Tech, China), Smad3 antibody (1:1,000 dilution, Protein-Tech, China), phospho-Smad3 (1:1,000 dilution, Protein-Tech, China), p62 (1:1,000 dilution, ProteinTech, China), α-tubulin (1:1,000 dilution, Affinity, China). The proteins were separated using SDS-PAGE and transferred onto a membrane, followed by incubation with horseradish peroxidase-conjugated anti-IgG (1:5000; Santa Cruz Biotechnology, Dallas, TX, USA). Protein bands were visualized using an enhanced chemiluminescence kit (Tanon; Tanon Science & Technology, Shanghai, China). To ensure accurate quantification, the Results were normalized with GAPDH (1:2000; Abcam).

### In vitro migration of lung fibrotic cells

To analyze the migratory and invasive properties of lung fibrotic cells, wound healing and transwell migration assays were performed, based on previously published methods^27^. To assess the migration capability of lung fibrotic cells, cell migration experiments were performed using a 24-well transwell plate (PET membrane 8 μm pore sizes, Corning Incorporated Costar, Corning, NY). Lung fibrotic cells (2 × 10^5^) suspended in 200 μL serum-free medium were seeded in the upper well of the transwell chambers, individually. In each group, one bottom was filled with 600 μL 0.5% serum medium as control, while in the experimental group, the bottom was filled with 4 × 10^5^ MSCs suspended in the same medium. After 24 h of incubation, non-migratory cells on the upper surface of the membrane were removed with a cotton swab, and the cells on the lower surface were fixed with methanol and stained with 0.1% crystal violet for 20 min. The stained cells were observed and counted using a fluorescence inverted microscope (Olympus, Tokyo, Japan). 10 photographs were captured in a tenfold objective lens to count the number of stained cells migrating through the membrane pore.

### Histology

Mice were euthanized and their right lungs were embedded in paraffin and sectioned for H&E or Masson’s trichrome staining^29^. The left lung was either snap frozen in liquid nitrogen and used for RNA and protein isolation or used fresh for collagen quantification or cytometric analysis. Randomly selected areas (10–15 fields) from 5 μm thick lung sections were examined under a Nikon Eclipse 80i microscope (Nikon) at × 100 and × 200 magnification. For histologic quantification, the Ashcroft score was used in a blinded fashion^30^. The Ashcroft score ranges from 0 to 8, with higher scores indicating more severe fibrosis. Scores of 0–1 represented no fibrosis, scores of 2–3 represented minimal fibrosis, scores of 4–5 were considered as moderate fibrosis, and scores of 6–8 indicated severe fibrosis.

### Sircol collagen assay

Collagen quantification was performed using the Sircol collagen assay following the manufacturer’s protocol (Bicolor, Life Science Assays)^31^. Briefly, the left lung was homogenized overnight at 4 °C in 5 mL of 0.5 M acetic acid with 0.6% v/v pepsin. A total of 1 mL of dye reagent was added to 100 μL of transparent supernatant, and the samples were vortexed for 30 min. The residual pellet was washed by acid-salt wash buffer to eliminate unbound collagen, and the pH was normalized with alkalization buffer. Absorbance was measured at a wavelength of 550 nm in a microplate reader. The collagen content was determined by comparing it to a standard curve, represented as mg/mL of left lung homogenate.

### Statistical analysis

All experimental data were expressed as the means ± standard deviation (SD). Statistical analysis was performed using the Student's independent t-test via Prism 9 software (GraphPad Software, La Jolla, CA, USA) between experimental groups. The significance level was set at *P < 0.05; **P < 0.01, ***P < 0.001, ****P < 0.0001.

## Results

### Extraction, differentiation, and identification of adipose-derived MSC

**Figure 1:**
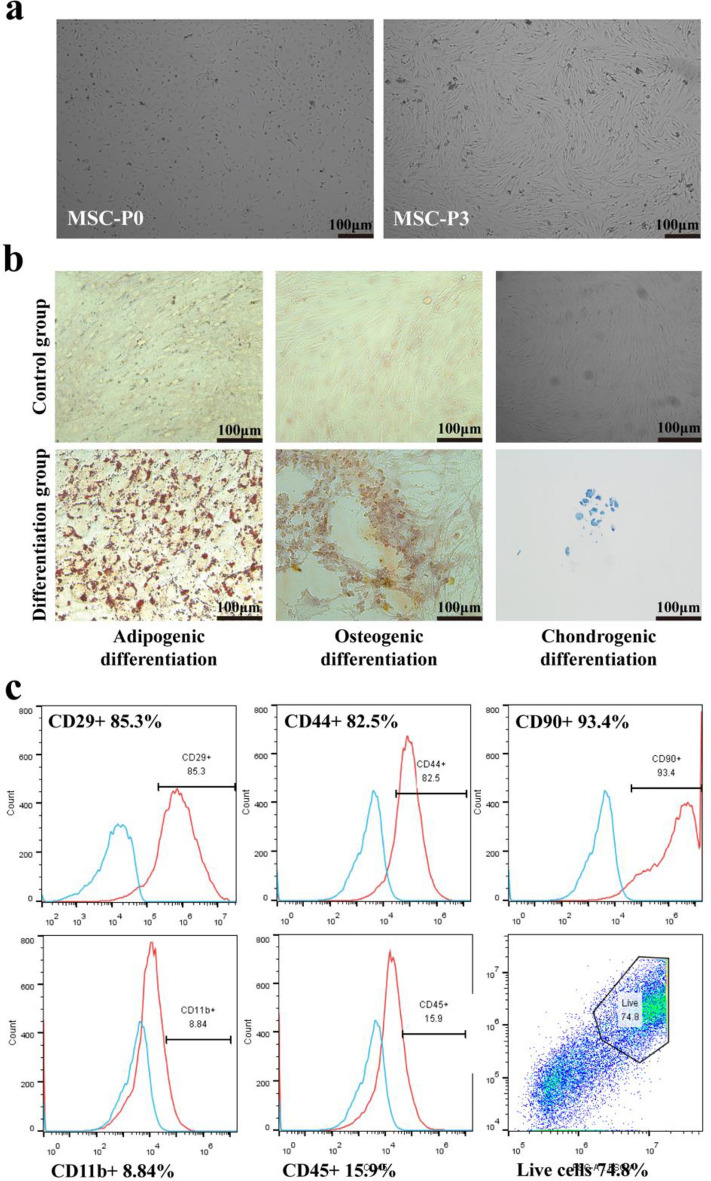
Cultivation, differentiation, and identification of MSCs. (**a**) Morphology of MSCs at different culture passages. The first-generation MSCs are generally triangular or dot-shaped, while the third-generation MSCs displayed a spindle-like or elongated shape. Images were taken at × 40 magnification. (**b**) MSCs subjected to differention and stained accordingly. Successfully differentiation into adipocytes (stained red with Oil Red), osteoblasts (stained with Alizarin Red), and chondrocytes (stained with Alcian Blue). Scale bar = 100 μm. Images were taken at × 100 magnification. (**c**) Flow cytometric analysis of MSCs after three passages of culture. The expression of CD45 and CD11b antigen/cell markers was found to be negative, while CD29, CD44, CD90, and other antibodies exhibited positive expression, Statistical analysis was performed using the Student’s t-test (two-tailed).

In this study, MSCs were isolated and cultured from adipose tissue in C57BL/6 mice. The sterile collection of adipose tissue from the epididymis was followed by digested with type I collagenase to remove impurities and mature adipocytes. The resulting primary adipose stem cells were subjected to adherent culture and passage to the third generation^32^. As the cells underwent adherent culture and passaging, their morphology gradually changed, showing a fibroblast-like morphology appearance characterized by a long spindle shape (Fig. 1a).

To assess the differentiation potential of the MSCs, specific differentiation media were employed.. In the differentiation into adipocytes experiment, the induced group exhibited the presence of large lipid droplets in the cytoplasm, which could be stained with oil red O, resulting in a red coloration. This observation indicated that the cells were capable of differentiating into adipocytes. In the osteogenic differentiation experiment, Alizarin Red staining revealed the formation of light brown calcium nodules in the induced group, suggesting successful differentiation into osteoblasts. Additionally, in the chondrogenic differentiation experiment, the induced MSCs formed cell aggregates and, upon slicing, exhibited a blue-green staining with Alcian Blue, indicating their ability to differentiate into chondrocytes (Fig. 1b).

Subsequently, flow cytometry analysis was conducted on the third-generation MSCs to confirm their phenotype (Fig. 1c). The results demonstrated negative expression of CD45 and CD11b , which are markers for hematopoietic cells and macrophages, respectively. Conversely, positive expression was observed for CD29, CD44, CD90, and other surface markers, consistent with the identification criteria outlined by the International Cell Association’s Identification criteria for adipose-derived MSCs.

### Bleomycin promotes lung fibrosis in mice

**Figure 2:**
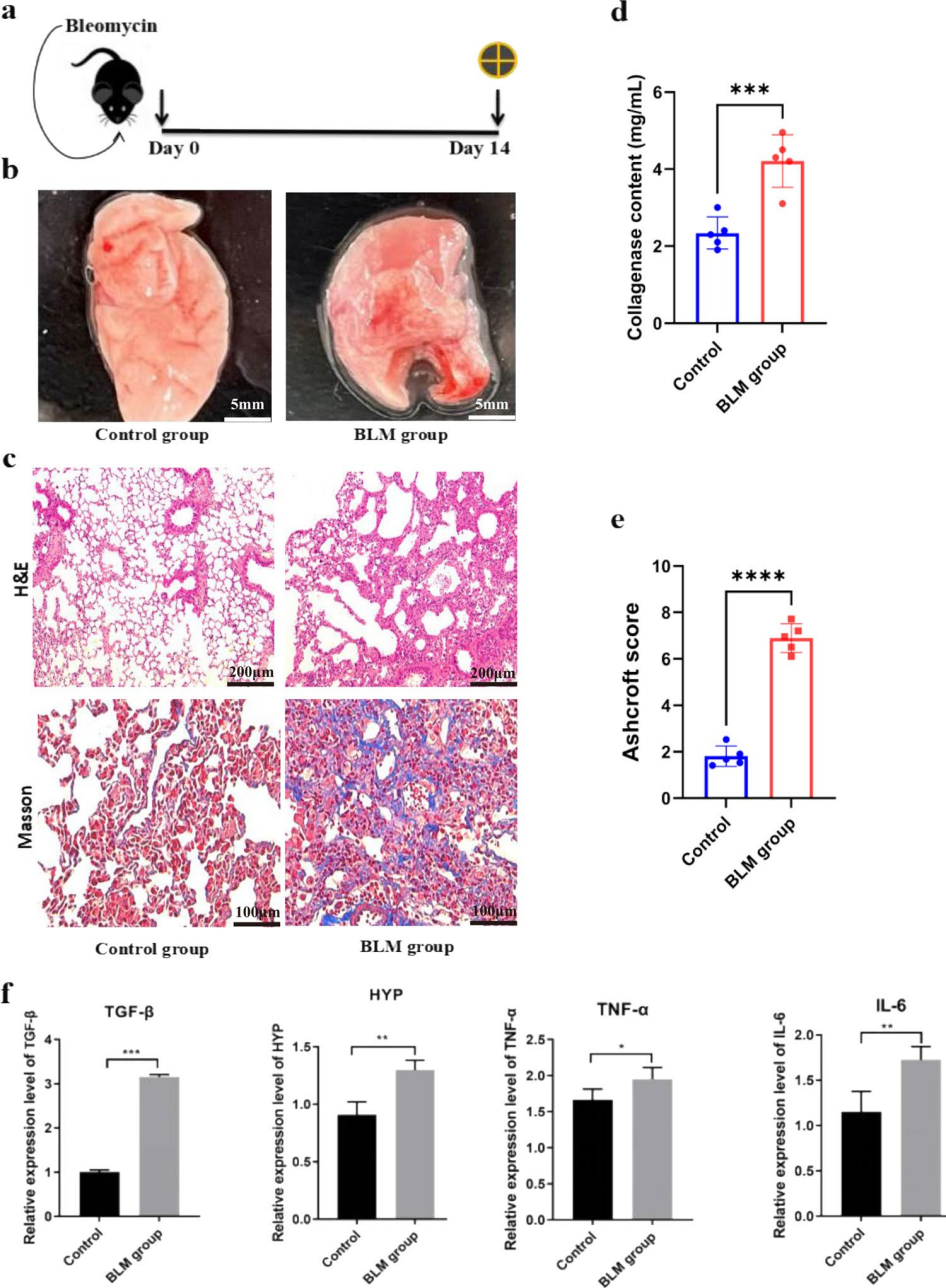
Bleomycin-induced pulmonary fibrosis mouse model. (**a**) Eight-week-old mice (C57BL/6 strain) received endotracheal bleomycin (3 U/kg) or 0.9% normal saline (control). on day 0. Mice were sacrificed on day 14. The cross symbol represents animal harvest. (**b**) Macroscopic observation of mouse lung tissue was conducted to assess the effects of the interventions. (**c**) Lung sections were stained with Hematoxylin–eosin and Masson’s trichrome. Images were taken at × 100 magnification. Bleomycin group showed architectural destruction, alveolar septal thickening, and fibrotic changes. Scale bar: 100/200 μm. (**d**) Collagen deposition was assessed by Sircol assay and represented as milligrams per milliliter (mg/mL) of left lung homogenate. (**e**) The extent of lung fibrosis was measured on day 14 by using the Ashcroft score. (**f**) qRT-PCR analysis of bleomycin induces changes in the expression of pulmonary fibrosis-related genes. The mice were sacrificed humanely, and lung tissues were collected to assess the mRNA levels of fibrosis-related genes. Expression of TGF-β, HYP, TNF-α, and IL-6 levels (n = 6 mice per group). Data are representative of 5 independent experiments, mean ± SD. n = 5 per experimental group; each symbol represents 1 mouse. *P < 0.05; **P < 0.01, ***P < 0.001, ****P < 0.0001, 1-way ANOVA followed by Fisher’s LSD post hoc analysis.

The therapeutic capacity of MSCs was assessed using a murine bleomycin-induced experimental model of pulmonary fibrosis to assess^33^. The mice in the study were exposed to bleomycin via nasal inhalation, while the control group received an equivalent volume of PBS solution (Fig. 2a). After three weeks, the success of the fibrosis model was evaluated by gross observation of lung tissue, stopathological analysis, and measurement of cytokine levels. Macroscopically, the lung tissues of the the drug group exhibited white spot-like material on the tissue surface and in the deeper tissue layers, while the the control group appeared smooth and free from lesions or necrosis (Fig. 2b).

Subsequently, lungs tissues were collected and subjected to hematoxylin–eosin (HE) staining (Fig. 2c). The control group displayed normal alveolar structure without obvious inflammation or fibrosis. In contrast, the drug group showed obvious deformation and hyperplastic alveolar structure, significantly widened alveolar spaces, thickened alveolar walls, edematous alveolar cavities, and extensive infiltration of inflammatory cells. Masson staining of lung tissues of the two groups revealed that the control group maintained a normal alveolar structure without obvious inflammation or fibrosis. However, the drug group exhibited severe showed severe hyperplasia, with prominent deposition of blue collagen fibers. Indicative of increased collagen content in bleomycin-induced pulmonary fibrosis(Fig. 2d). Consistently, compared to the control group, animals received bleomycin presented with a higher Ashcroft score , reflecting more severe fibrotic changes (Fig. 2e).

Furthermore, genomic RNA was extracted from lung tissue to assess the expression levels of relevant factors (Fig. 2f). The drug group exhibited significantly elevated expression of transforming growth factor β (TGF-β), hydroxyproline (HYP; which can indicate the degree of pulmonary fibrosis), Tumor Necrosis Factor α (TNF-α), and Interleukin 6 (IL-6) gene compared to the control group. These findings indicate that bleomycin administration led to the establishment of a stable pathological state of pulmonary fibrosis.

### MSC therapy reverses bleomycin-induced pulmonary fibrosis

**Figure 3:**
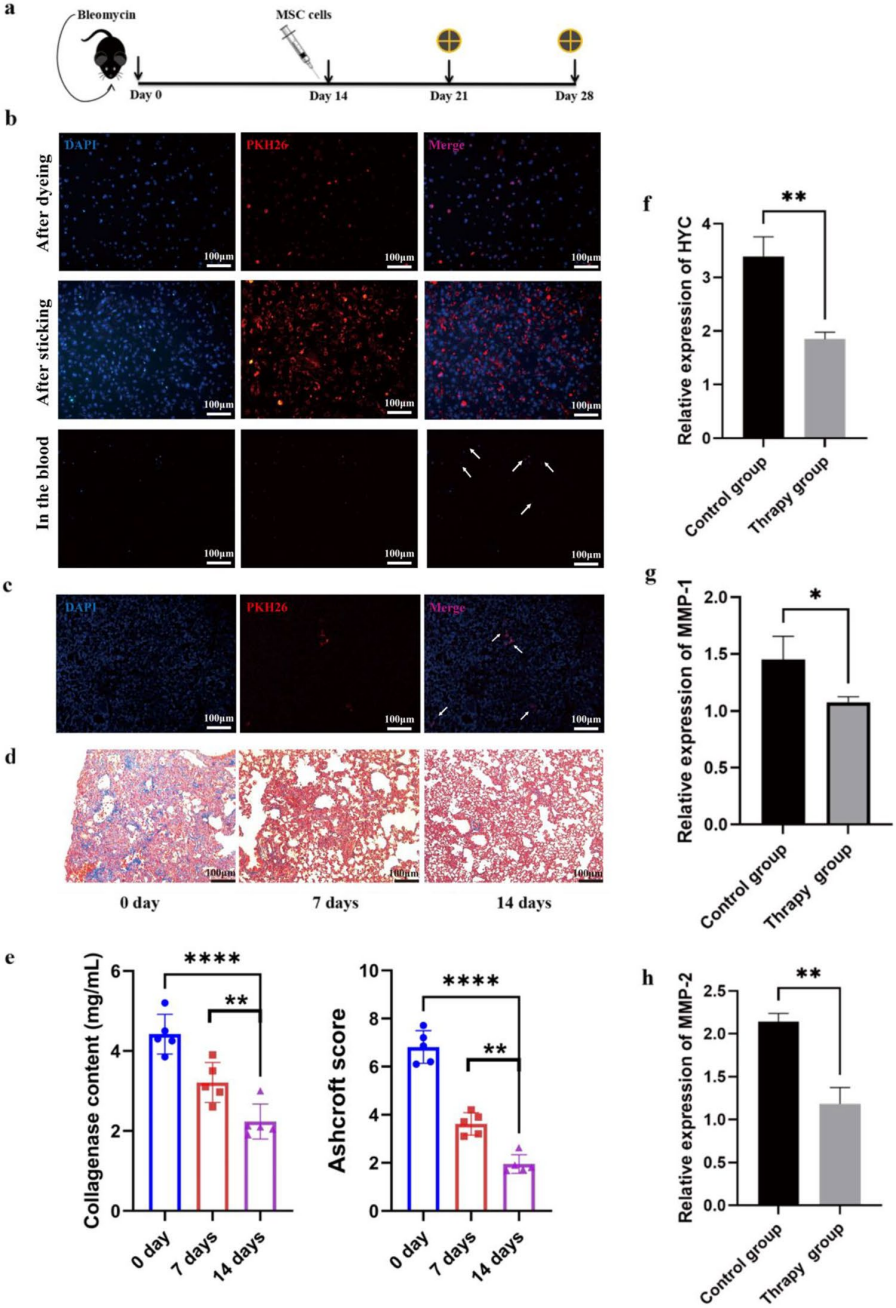
MSC therapy reverts bleomycin-induced pulmonary fibrosis. (**a**) Fourteen days after the administration of bleomycin, MSCs were administered, and mice were sacrificed on day 21 or 28 for further analysis. The cross symbol represents the time of animal harvest. (**b**) MSCs were labeled with fluorescent dyes, DAPI for the nucleus, and PKH26 for the cell membrane. After the mice were sacrificed, the presence of fluorescently labeled MSCs in their peripheral blood was detected. (**c**) Lung slices are used for fluorescence microscopy. The image was taken at × 100 magnification. Fluorescently labeled MSCs can be observed after cell therapy. (**d**) Lung sections were stained with Mason's trichrome. The image was taken at × 100 magnification. Collagen deposition decreased in a time dependent fashion following MSC therapy. Scale bar: 100 μm. (**e**) Collagen deposition was assessed by Sircol assay and the result were represented as mg/mL of left lung homogenate. The degree of lung fibrosis was measured using the Ashcroft score at days 0, 7 and 14 after treatment. (**f**–**h**) qRT-PCR analysis of gene expression of related cytokines in lung tissue after MSC cell therapy. The mice were sacrificed humanely, and lung tissues were collected to assess the mRNA levels of fibrosis-related genes. Expression of HYP, MMP-1, and MMP-2 levels (n = 6 mice per group). Data are representative of 5 independent experiments, mean ± SD. n = 5 per experimental group; each symbol represents 1 mouse. *P < 0.05; **P < 0.01, ***P < 0.001, ****P < 0.0001, 1-way ANOVA followed by Fisher’s Dunnett post hoc analysis.

In this study, the therapeutic potential of MSCs was evaluated in a murine model of bleomycin-induced pulmonary fibrosis (Fig. 3a). In this study, the therapeutic potential of MSCs was evaluated in a murine model of bleomycin-induced pulmonary fibrosis DAPI and PKH26 fluorescent dyes were utilized to label the cell. After three days, peripheral blood samples were collected from the mice and examined under a fluorescence microscope. Fluorescently labeled active MSCs were present in the bloodstream, indicating their ability to circulate throughout the body (Fig. 3b).

Following MSCs injection, lung tissues were harvested at 7 and 14 days and analyzed through frozen sections under a fluorescence microscope. The presence of MSCs was observed in the lung samples, indicating their migration to lung tissue upon administration, thus demonstrating their potential for therapeutic intervention (Fig. 3c).

To assess the efficacy of MSCs therapy in pulmonary fibrosis mice, lung tissues from mice treated with MSCs were subjected to Masson staining. The results demonstrated a significant reduction in the lung tissues after one week of cell treatment. Over time, the presence of blue collagen tissues gradually decreased, indicating a progressive decrease in collagen deposition (Fig. 3d). These findings highlight the ability of MSCs could to reduce the level of collagen accumulation, alleviate lung fibrosis, and promote the restoration of lung tissue function. Concurrently, the animals treated with MSCs exhibited a gradual decrease in the Ashcroft score, further supporting the beneficial effects of MSC therapy (Fig. 3e).

Subsequently, the expression of lung fibrosis-related factors was examined in both the control group and the cell therapy group. The gene expression of hydroxyproline (HYP), matrix metalloproteinase-1 (MMP-1), and matrix metalloproteinase-2 (MMP-2) were significantly lower in the cell therapy group compared to the control group. These findings indicate that the presence of MSCs can reverse the pathological state of pulmonary fibrosis , reduce the level of fibrotic lesions, and modulate the expression of key factors involved in fibrosis development (Fig. 3f–h).

### Construction of a cellular model of pulmonary fibrosis

**Figure 4:**
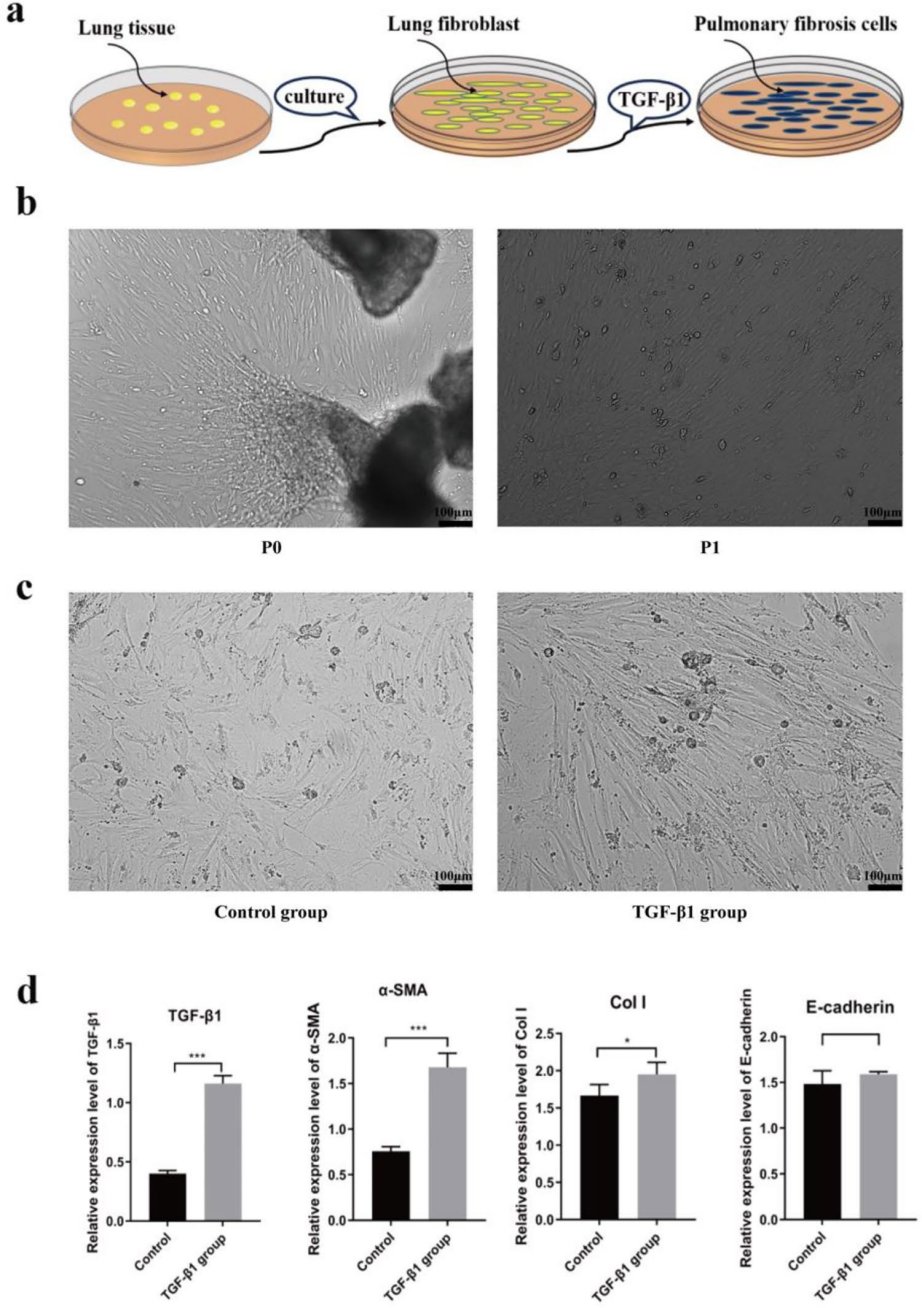
The cellular model of pulmonary fibrosis induced by TGF-β1. (**a**) Pulmonary fibroblasts were isolated from lung tissue using an adherent culture method, and subsequently, TGF-β1 was employed to induce their transformation into pulmonary fibrotic cells. (**b**) Pulmonary fibroblasts were purified from lung tissue and cultured for cell passage. Images were taken at × 40 magnification. Scale bar: 100 μm. (**c**) Following treatment with TGF-β1 (10 mg/L), lung fibroblasts underwent induction and acquired a fibrotic phenotype. Images of the induced cells were taken at × 40 magnification. Scale bar: 100 μm. (**d**) qRT-PCR analysis of gene expression supports the formation of a cellular model of pulmonary fibrosis following induction by TGF-β1. Expression of TGF-β1, α-SMA, Col I and E-cadherin levels (n = 3 per group). Data are representative of 5 independent experiments, mean ± SD. n = 5 per experimental group. *P < 0.05; **P < 0.01, ***P < 0.001, 1-way ANOVA followed by Fisher’s LSD post hoc analysis.

The therapeutic potential of MSCs in reversing pulmonary fibrosis has been demonstrated in mice. Additionally, this paper investigated the effects of MSCs on pulmonary fibrosis cells at the cellular level. Initially, pulmonary fibroblasts were isolated from lung tissue using an adherent culture method. Subsequently, these fibroblasts were induced into pulmonary fibrotic cells using TGF-β1 (Fig. 4a). A substantial number of fibroblasts were successfully obtained from the lung tissue of newborn C57 mice , as depicted in Fig. 4b. Following the treatment with TGF-β1 (10 mg/L), the morphology of the lung fibroblasts retained a long spindle-shaped state without signs ofaging (Fig. 4c).

To confirm the establishment of the pulmonary fibrosis cell model, this study assessed the expression levels of relevant factors.. The results revealed a significant upregulation of related genes in the TGF-β1 -treated group compared to the control group (Fig. 4d). These genes included transforming growth factor β1 (TGF-β1), α-smooth muscle actin (α-SMA), and type I collagen. However, there were no significant differences between the groups in terms of E-cadherin content. This findings indicate that treatment of lung fibroblasts with TGF-β1 induces intracellular collagen deposition and fibrotic alterations.

### MSC supernatant promotes the migration and proliferation of fibrotic lung cells in vitro

**Figure 5:**
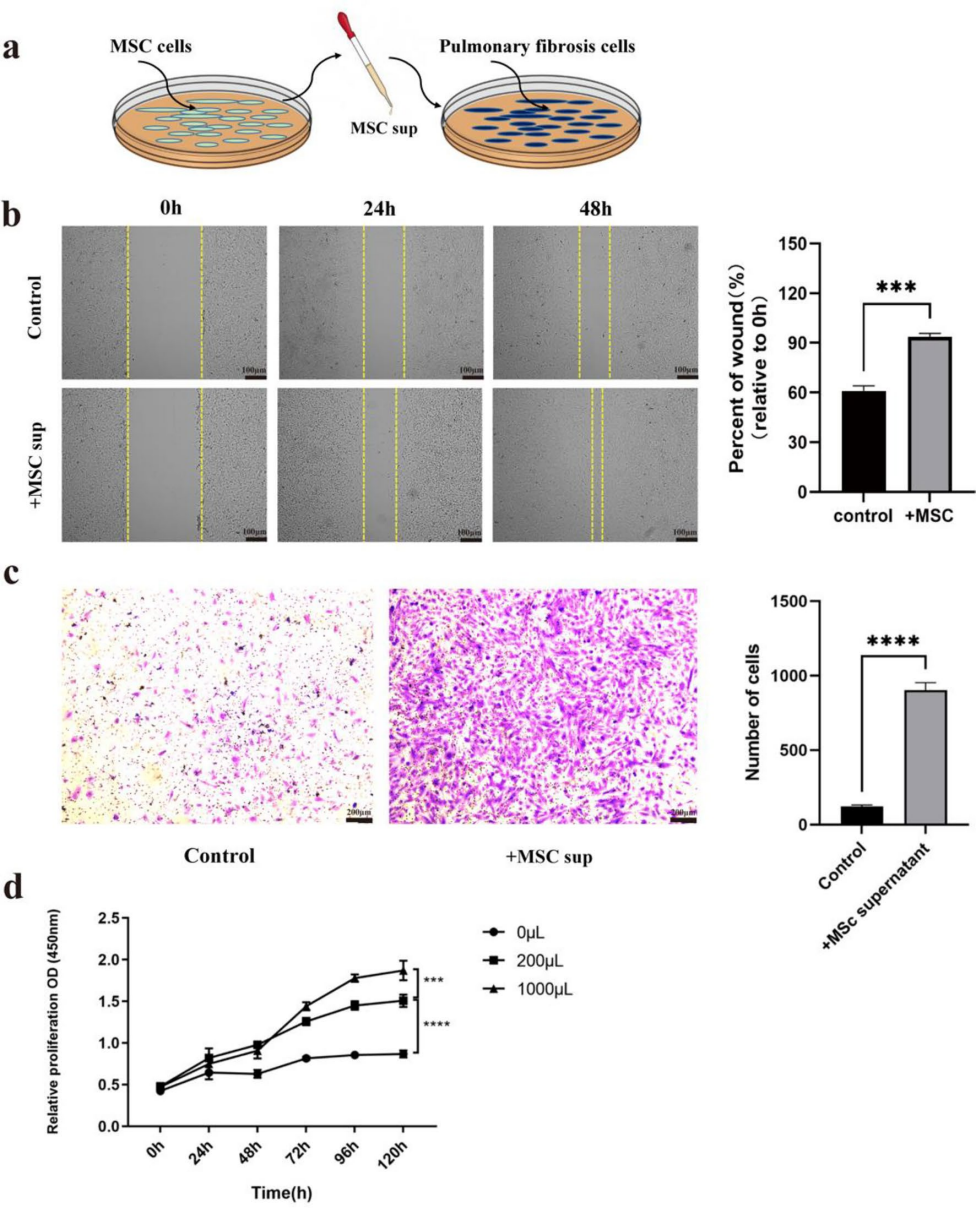
MSC supernatant promotes the migration and proliferation of fibrotic lung cells in vitro. (**a**) MSC supernatant was harvested and added to the culture medium of lung fibrosis cells. (**b**) The migration level of lung fibrotic cells was evaluated through a wound healing experiment following the addition of MSC supernatant. The results of this experiment provided insights into the migratory capacity of the cells upon exposure to MSC supernatant. (**c**) The transwell migration assay method detects the migration level of lung fibrotic cells after adding MSC supernatant. (**d**) The CCK-8 viability assay of the lung fibrotic cells added to the MSC supernatant. All results are represented as mean ± SD. n = 5 per experimental group. ***P < 0.001, ****P < 0.0001, 2-way ANOVA followed by Fisher’s Dunnett post hoc analysis.

Following the successful establishment modeling of the pulmonary fibrosis cell model, the impact of MSCs supernatant on the migration and proliferation of these cells was t investigated in this study. (Fig. 5a). To assess the effect of MSC supernatant on the migration of lung fibrotic cells, a cell wound healing experiment was conducted. The results revealed a significant increase in cell migration upon addition of MSC supernatant (Fig. 5b). These findings suggest that the cytokines secreted by MSCs into the supernatant may exert a positive influence on fibrotic lung cells.

A transwell assay was employed to further examine the effect of MSC supernatant on the migratory potential of fibrotic lung cells (Fig. 5c). Notably, the number of cells passing through the membrane increased significantly following exposure to MSC supernatant. This observation suggests that MSCs possess a certain tropism towards pulmonary fibrotic cells and can promote their migration.

Finally, to investigate the impact of MSC supernatant on the proliferation of fibrotic lung cells, a CCK-8 assay was performed. As the culture duration extended, the addition of MSC supernatant resulted in a significantly increase in the optical density (OD) value. Furthermore, the promotion of cell proliferation by MSC supernatant exhibited a dose-dependent pattern (Fig. 5d).

### MSCs promote the migration and proliferation of lung fibrotic cells in vitro

**Figure 6:**
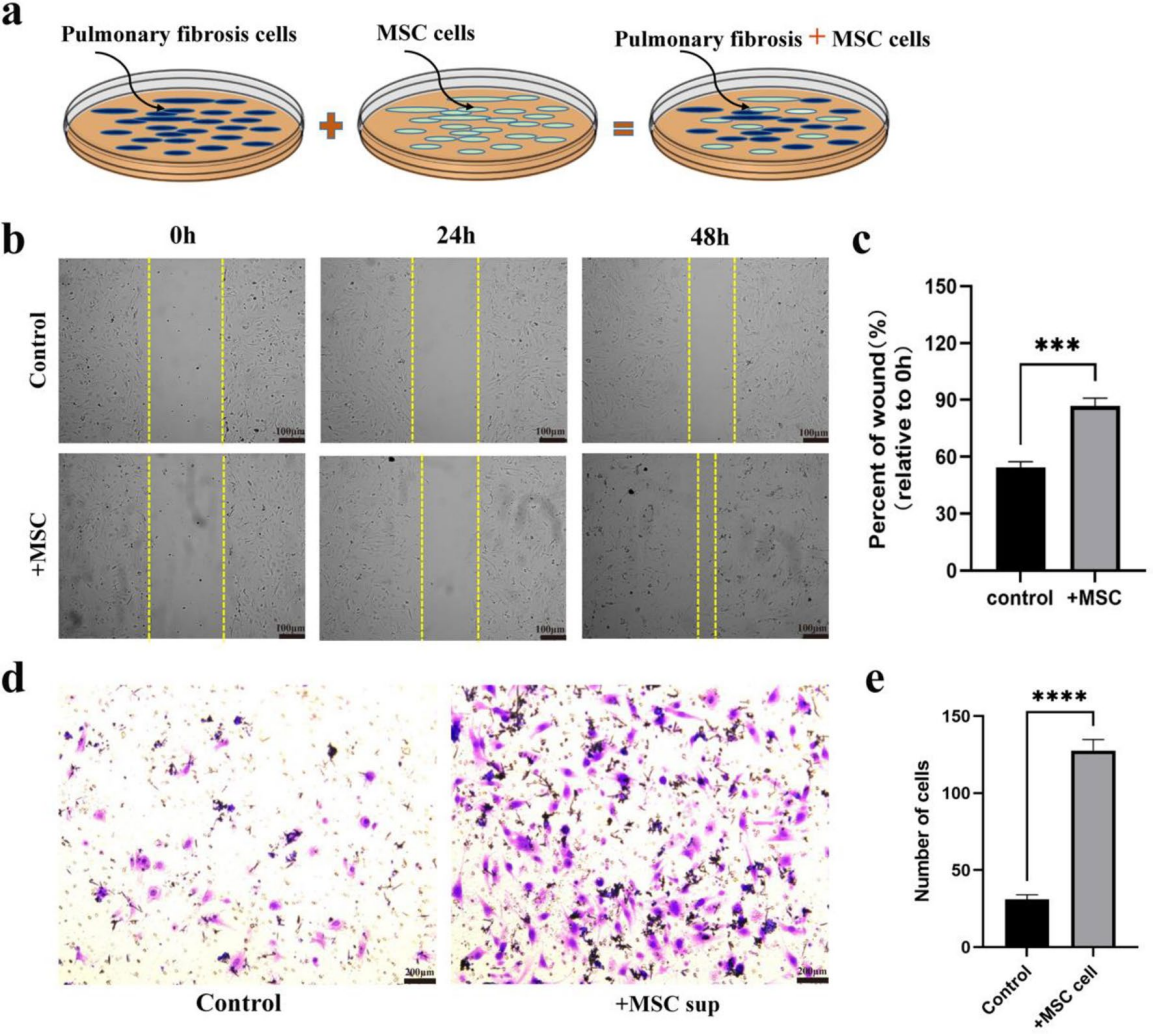
MSCs promotes the migration and proliferation of lung fibrotic cells in vitro. (**a**–**c**) Promotion of Migration and Proliferation of Lung Fibrotic Cells by MSCs In Vitro. (**a**) Co-culture of MSCs and lung fibrotic cells. MSCs were harvested and added to the culture medium of lung fibrotic cells. (**b**,**c**) Wound healing assay. (**d**,**e**) Transwell invasion assay. All results are represented as mean ± SD. n = 5 per experimental group. ***P < 0.001, ****P < 0.0001,1-way ANOVA followed by Fisher’s LSD post hoc analysis.

To explore the effect of MSCs on lung fibrotic cells, a direct co-culture model was established. Lung fibrotic cells were co-cultured with MSCs to investigate their impact on the biological function of lung fibrotic cells. The two types of cells are mixed and cultured at a ratio of 1:1(Fig. 6a). Cell wound healing experiments showed that a co-culture of MSCs and lung fibrotic cells assess the rate of wound closure in the co-culture system compared to the control group (Fig. 6b,c). These results provide evidence for the potential of MSCs to accelerate wound healing in lung fibrotic cells.

The lower chamber of the Transwell system contained MSCs, while the upper chamber contained lung fibrotic cells. The number of lung fibrotic cells passing through the chamber increases significantly, showing that MSCs can promote the migration level of lung fibrotic cells (Fig. 6d,e).

In summary, this study provides evidence that MSCs enhance the migration and proliferation of lung fibrotic cells in vitro. Co-culture experiments demonstrate an accelerated rate of wound healing in the presence of MSCs. Furthermore, Transwell assays confirm the ability of MSCs to promote the migration of lung fibrotic cells. These findings shed light on the potential therapeutic applications of MSCs in lung fibrosis treatment. Further investigations are warranted to elucidate the underlying mechanisms and validate these findings in vivo.

### MSC supernatant attenuates TGF-β1-induced fibroblast activation

Finally, this study investigated the effects of MSCs supernatant on the expression levels of fibrosis -related genes and proteins in pulmonary fibrosis cells.

**Figure 7:**
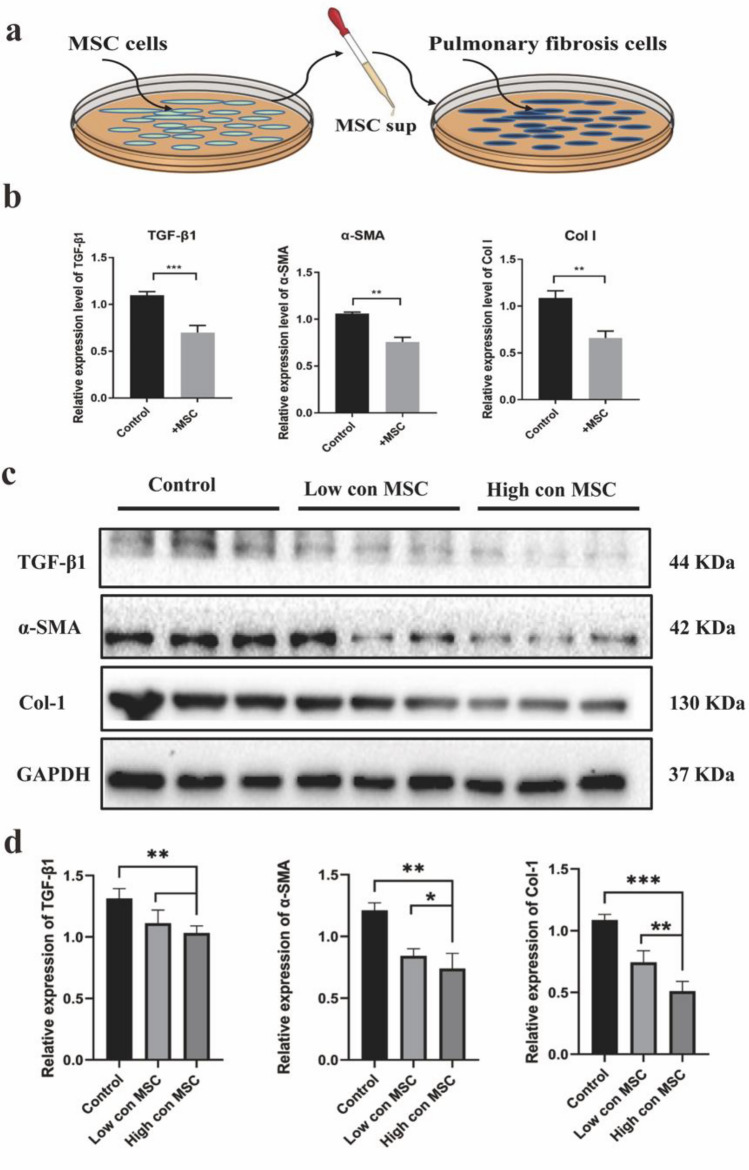
MSCs supernatant reduces the expression of fibrosis-related genes and protein levels of pulmonary fibrosis cells. (**a**) The MSC supernatant was harvested and added to the culture medium of lung fibrosis cells. (**b**) qRT-PCR analysis showed that the application of MSC supernatant resulted in the reversel of pulmonary fibrotic cells formation induced by TGF-β1. The expression levels of TGF-β1, α-SMA, and collagen type I were assessed (n = 3 per group). (**c**,**d**) Western blot analysis was performed to evaluate the relative protein levels at different doses of MSC supernatant. The expression levels of TGF-β1, α-SMA, and Col I levels (n = 3 per group). Data are representative of 5 independent experiments, mean ± SD. n = 5 per experimental group. *P < 0.05; **P < 0.01, ***P < 0.001, 1-way ANOVA followed by Fisher’s LSD post hoc analysis. Original blots are presented in Supplementary Fig. S3.

Firstly, The MSCs supernatant was collected and added to the culture medium of lung fibrosis cells in low (1 mL sup to 9 mL culture medium) and high (5 mL sup to 5 mL culture medium) concentration (Fig. 7a). To assess the impact on gene expression, this study used qRT-PCR analysis to detect the gene levels of pulmonary fibrosis-related factors. The results revealed a significant reduction in the gene levels of TGF-β1, α-SMA, and type I collagen in the MSC supernatant -treated group compared to the control group. This suggests that the MSC supernatant has the ability to modulate collagen deposition in pulmonary fibrotic cells, potentially through the secretion of active substances by MSCs that improve fibrosis (Fig. 7b).

Next, this paper examined the protein levels of pulmonary fibrosis-related factors using Western blot analysis. Lung fibroblasts were treated with different concentrations of MSC supernatant, This study used high and low concentrations of MSC supernatant to treat lung fibroblasts, and observed a dose-dependent decrease in the protein expression levels of TGF-β1, α-SMA and, type I collagen (Fig. 7c,d).

Western blot assay was performed to explore whether MSC supernatant could regulate the TGF-β1/Smad signaling pathway. As shown in Supplementary Fig. 1, MSC supernatant reduced the proportions of pSmad2 to Smad2 and p-Smad3 to Smad3 in fibroblasts cells. These data revealed that MSC supernatant could suppress the TGF-β1/Smad signaling pathway to inhibit the TGF-β1-induced fibroblast activation. The MSC supernatant evidently reduced the CQ- and the Baf A1-induced protein expression levels of p62 (Supplementary Fig. 2), which indicated that MSC supernatant could promote autophagy.

## Discussion

According to recent reports, only Pirfenidone and Nintedanib are used to delay lung function decline but cannot elongate the life span of IPF patients. Currently, lung transplantation is the only effective treatment strategy, but IPF remains a great challenge due to its complexity and limited availability of donor organs^34^.

Studies show that MSCs and MSCs supernatant have a therapeutic potential on the BLM-induced lung fibrosis. MSCs have emerged as a promising therapeutic option for IPF.^35^. Here, we firstly evaluated the role of MSCs on pulmonary fibrosis. According to an official American Thoracic Society workshop report, murine intratracheal BLM model is the bestcharacterized animal model available for preclinical experiments of pulmonary fibrosis^36^. Thus, this study used the BLM to establish the in vivo pulmonary fibrosis model. Meanwhile, MSCs were successfully isolated and cultured from adipose tissue^37^. MSCs were then injected into mice through the tail vein. In this study, MSCs were found to reside in the blood of mice for two weeks and were able to migrate to lung tissue. The hydroxyproline content in the total lung sample is a substitute for the collagen content (1 mg of hydroxyproline = 6.94 mg of collagen) and is the gold standard for assessing the severity of pulmonary fibrosis^38^. In this study, MSCs could significantly decrease the level of hydroxyproline in the BLM-injured mice. Pulmonary function is a key indicator of treatment efficiency in clinical trials and can also be used to confirm fibrosis changes and treatment response to interventions in animal models^39^. This study also observed the significant improvement of pulmonary function in bergenin-treated mice. Consistent with the results of hydroxyproline experiments, the Masson's trichrome staining results also showed that MSCs could significantly alleviate BLM-induced pulmonary fibrosis in mice.

The activation and proliferation of fibroblasts leads to the progression of fibrosis and disrupts the normal structure and function of the lung tissue. Therefore, elimination of excess fibroblasts can be a strategy for the treatment of fibrosis^40^. TGF-β/Smad signaling is the main pathway of fibrogenesis, including IPF. Activation of TGF-β1/Smad signaling pathway leads to myofibroblast activation, overproduction of ECM, and inhibition of ECM degradation^41^. The typical activation marker of lung fibroblasts is α-smooth muscle actin (α-SMA) and ECM deposition mainly reflected in the overexpression of type collagen I (Col-I)^42^. This study chose to treat fibroblasts with TGF-β1 to establish in vitro models, and evaluate the expression of α-SMA and Col-I. This study found that the presence of MSC or its supernatant could promote the proliferation and migration of lung fibrotic cells, which consistent with previous reports. Additionally, the present study measured the expression of α-SMA and Col-I in the in vitro model. And the results indicated that MSC supernatant could effectively reduce the content of lung fibrosis-related substances.

MSCs possess the capability to secrete a variety of cytokines and growth factors, including TGF-β, HGF, and VEGF, which have demonstrated their potential in promoting tissue repair and regeneration^43^. In this study, the therapeutic effects of MSCs were likely mediated through the secretion of TGF-β1. TGF-β1 is a cytokine that plays a critical role in the pathogenesis of pulmonary fibrosis^44^. It can induce the differentiation of fibroblasts into myofibroblasts, which are the primary effector cells in the development of pulmonary fibrosis^45^. Remarkably, this study revealed a significant reduction in the expression of TGF-β1 in lung fibroblasts upon treatment with the MSCs supernatant. i This observation strongly suggests that the anti-fibrotic effects associated with TGF-β1 signaling pathways. TGF-β1 signaling pathway significantly influences the regulation of fibroblast activation. The Smad2 and Smad3 are major downstream regulators that promote the TGF-β1-mediated lung fibrosis^46^. Mechanism study found that MSCs supernatant could inhibit TGF-β1-induced phosphorylation of Smad2 and Smad3 to suppress the activation of pulmonary fibrosis cells. These results are in line with previous reports, which suggest that MSCs have anti-fibrotic properties through the secretion of various cytokines^47,48^.

A decrease in the autophagy flux is observed in the lung tissue homogenates of patients with IPF^49^. In this sense, the induction of the fibroblast autophagy may be beneficial for pulmonary fibrosis. The p62, an autophagy adaptor protein, which could bind ubiquitylated protein and delivering them to the autophagosomes. Autophagy is deficient in patients with IPF, and TGF-β1 can inhibit the autophagy activation^50^. This study found that MSCs supernatant downregulates the protein level of p62 in the presence of CQ or Baf A1, revealing that MSCs supernatant may promote autophagy. The autophagy and apoptotic processes have a complex relationship and jointly dominate the fate of the cell. Autophagy can reduce the pathological progression of IPF by promoting fibroblast apoptosis, and inhibition of autophagy can interfere with the induction of apoptosis^51^. This study explored that MSCs supernatant could could maintain normal autophagy by downregulating the protein level of p62. However, the specific targets need further study.

Although the use of MSCs or MSCs supernatant in chronic lung diseases, including IPF, is promising, there are limitations to their use that raise safety concerns^52^ Such problems that create limitations are the selection and processing of cell grafts, the use of allogeneic versus autologous MSCs, the route of administration, and the rare but present possibility of carcinogenesis^53^. Thus, MSCs supernatant could be used for treating pulmonary fibrosis, mainly IPF, as a cell-free therapy with the advantages mentioned earlier compared to MCSs therapies. In addition, the intravenous administration of MSCs supernatant therapies makes them easier to use compared to stem cell therapies.

## Conclusion

In summary, this study provides further evidence for the potential therapeutic application of MSCs/supernatant in the treatment of pulmonary fibrosis. The current study has demonstrated that MSCs supernatant alleviates the BLM-induced pulmonary fibrosis in vivo. In vitro experiments further reveal that MSC supernatant could suppress the TGF-β 1/Smad signaling pathway to inhibit the TGF-β1-induced fibroblast activation, and promotes fibroblast autophagy by Regulating p62 expression. However, the current study have not confirmed the specific target of MSCs supernatant yet. Further studies are needed to investigate the target of MSCs supernatant and whether it affects other signal pathways related with pulmonary fibrosis should be evaluated.

### Supplementary Information

Supplementary Information.

## Data Availability

The datasets generated during and/or analyzed during the current study are available from the corresponding author on reasonable request.
