# Supplementary material for: Adipose-derived mesenchymal stem cell therapy for reverse bleomycin-induced experimental pulmonary fibrosis
Source: Sci Rep. 2023 Aug 14;13:13183. doi: 10.1038/s41598-023-40531-9 (PMC10425426; doi:10.1038/s41598-023-40531-9)
Supplement: Supplementary file 1 — Supplementary Information. [file 41598_2023_40531_MOESM1_ESM.docx]

**Supplementary material**

**Adipose-derived Mesenchymal Stem Cell therapy for reverse bleomycin-induced experimental pulmonary fibrosis**

Xiansheng Zhao, Jinyan Wu, Ruoyue Yuan, Yue Li, Quyang Yang, Baojin Wu, Xiaowen Zhai, Jiucun Wang, Jérémy Magalon, Florence Sabatier, Aurélie Daumas, Winston M Zhu, Ningwen Zhu*.

**MSC supernatant suppresses TGF- β1-induced activation of** **Smad signaling pathway**

TGF- β1 signaling pathway significantly influences the regulation of fibroblast activation. The Smad2 and Smad3 are major downstream regulators that promote the TGF- β1-mediated lung fibrosis. Western blot assay was performed to explore whether MSC supernatant could regulate the TGF- β1/Smad signaling pathway. As shown in **Supplementary Figure 1**, MSC supernatant reduced the proportions of pSmad2 to Smad2 and p-Smad3 to Smad3 in fibroblasts cells. These data revealed that MSC supernatant could suppress the TGF- β1/Smad signaling pathway to inhibit the TGF- β1-induced fibroblast activation.


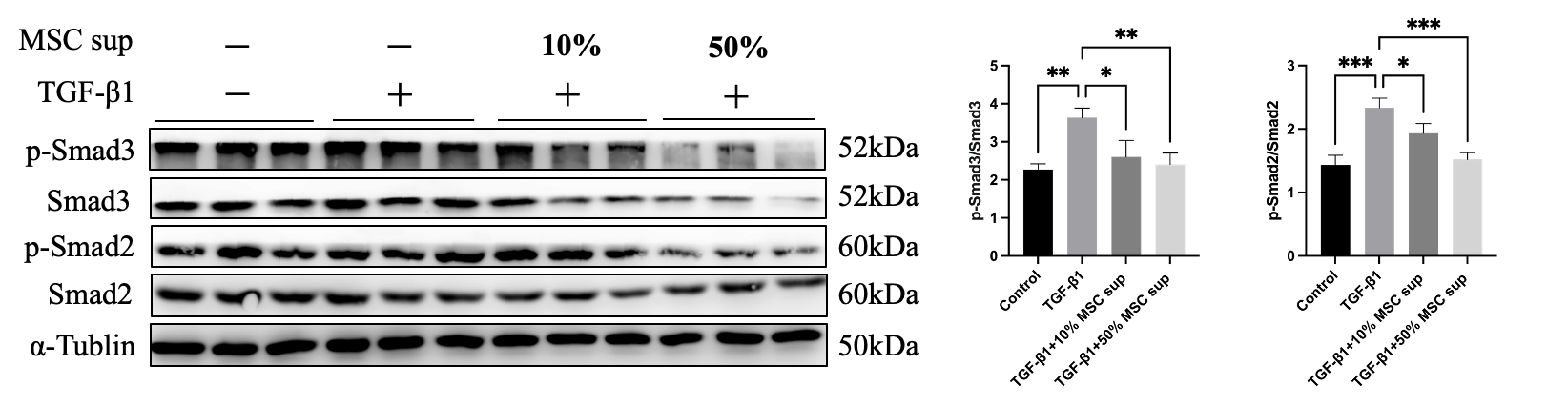


**Supplementary Figure1.** **MSC supernatant suppresses TGF- β1-induced activation of Smad signaling pathway.**

Fibroblasts cells were pretreated with MSC supernatant (10% and 50% concentrations) for 24 h then incubated with TGF- β1 (10 mg/L) for 30 min. The expression of p-Smad3 and p-Smad2 in Fibroblasts cells. α-Tublin was used as the internal control. Data are presented as the means ± SD. Experiments were performed in triplicate (n = 3). *p < .05; **p < .01; ***p < .001. Original blots are presented in Supplementary Fig. S4.

**MSC supernatant promotes fibroblast autophagy by Regulating p62 expression**

Autophagy is deficient in patients with IPF, and TGF- β1 can inhibit the autophagy activation. The effect of MSC supernatant on the fibroblast autophagy was investigated. At first, the cellular model of inhibitory autophagy was established. Chloroquine (CQ) and Bafilomycin A1 (Baf A1) were two autophagy inhibitors that could increase the expression levels of p62. CQ and Baf A1 were used to inhibit autophagy in fibroblasts cells before treatment with MSC supernatant. The MSC supernatant evidently reduced the CQ- and the Baf A1-induced protein expression levels of p62 (**Supplementary Figure 2**), which indicated that MSC supernatant could promote autophagy.

**Supplementary Figure2. MSC supernatant promotes fibroblast autophagy by Regulating p62 expression.** Fibroblasts cells were treated with CQ (20 μM, **Fig2a**) and Baf A1 (100 nM, **Fig2b**) with or without MSC supernatant (10% and 50% concentrations) for 24 h. Protein levels of p62 in fibroblasts cells, GAPDH was used as the internal control. Western blots were cropped prior to incubation with primary antibody hybridization. Experiments were performed in triplicate (n = 3). *p < .05; **p < .01; ***p < .001. Original blots are presented in Supplementary Fig. S5.

**Supplementary Figure 3.** **Original Western blot blots of Figure 7c**

Western blots were cropped prior to incubation with primary antibody hybridization.

**Supplementary Figure 4. Original Western blot blots of Supplementary Figure1**

Western blots were cropped prior to incubation with primary antibody hybridization.

**Supplementary Figure 5. Original Western blot blots of Supplementary Figure2**

Western blots were cropped prior to incubation with primary antibody hybridization.
